# Medicinal plant knowledge of the Bench ethnic group of Ethiopia: an ethnobotanical investigation

**DOI:** 10.1186/1746-4269-5-34

**Published:** 2009-11-13

**Authors:** Mirutse Giday, Zemede Asfaw, Zerihun Woldu, Tilahun Teklehaymanot

**Affiliations:** 1 Aklilu Lemma Institute of Pathobiology, PO Box 1176, Addis Ababa University, Addis Ababa, Ethiopia; 2 The National Herbarium, Addis Ababa University, PO Box 3434, Addis Ababa, Ethiopia

## Abstract

**Background:**

Plants have traditionally been used as a source of medicine in Ethiopia since early times for the control of various ailments afflicting humans and their domestic animals. However, little work has been made in the past to properly document and promote the knowledge. Today medicinal plants and the associated knowledge in the country are threatened due to deforestation, environmental degradation and acculturation. Urgent ethnobotanical studies and subsequent conservation measures are, therefore, required to salvage these resources from further loss. The purpose of the present study was to record and analyse traditional medicinal plant knowledge of the Bench ethnic group in Southwest Ethiopia.

**Methods:**

Semi-structured interviews were conducted with Bench informants selected during transect walks made to houses as well as those identified as knowledgeable by local administrators and elders to gather data regarding local names of medicinal plants used, parts harvested, ailments treated, remedy preparation methods, administration routes, dosage and side effects. The same method was also employed to gather information on marketability, habitat and abundance of the reported medicinal plants. Purposive sampling method was used in the selection of study sites within the study district. Fidelity Level (FL) value was calculated for each claimed medicinal plant to estimate its healing potential.

**Results:**

The study revealed 35 Bench medicinal plants: 32 used against human ailments and three to treat both human and livestock ailments. The majority of Bench medicinal plants were herbs and leaf was the most frequently used part in the preparation of remedies. Significantly higher average number of medicinal plants was claimed by men, older people and illiterate ones as compared to women, younger people and literate ones, respectively. The majority of the medicinal plants used in the study area were uncultivated ones.

**Conclusion:**

The study revealed acculturation as the major threat to the continuation of the traditional medical practice in the study area. Awareness should, therefore, be created among the Bench community, especially the young ones, by concerned organizations and individuals regarding the usefulness of the practice.

## Background

A study showed that nearly 80% of the Ethiopian population is still dependent on traditional medicine, which largely involves the use of plants [1]. However, little effort has so far been made to document medicinal plants and the associated knowledge despite ongoing deforestation, environmental degradation and acculturation in the country. Hence, there is a need for immediate ethnobotanical surveys to save medicinal plants and the associated knowledge from further loss.

The purpose of this ethnobotanical study was to document and analyse medicinal plant knowledge of the Bench ethnic group. Bench, the language of the Bench ethnic group, belongs to the greater Omotic languages family [2]. The Bench are among the major ethnic groups inhabiting the Bench-Maji Zone, the Southern Nations, Nationalities and Peoples' Regional State (SNNPRS) in southwest Ethiopia. The other major ethnic groups in the Bench-Maji Zone are the Meinit, Sheko, Surma, Dizi and Majangir. Mizan Teferi (7° 04' N, 35° 30' E) is the administrative town of the Zone and is located at 561 km southwest of the Ethiopian capital, Addis Ababa. The majority of the Bench people live in Bench, one of the nine administrative districts (weredas) in the Bench-Maji Zone. Part of the Bench population also resides in the neighbouring Shey Bench District. In 1994, the population of the Bench ethnic group was estimated at 173,149 [3]. The Bench people are subsistence farmers who cultivate *Zea mays *L., *Sorghum bicolor *(L.) Moench and some root crops such as *Colocasia esculenta *(L.) Schott and *Dioscorea *spp. as their major staple crops. *Coffea Arabica *L. and *Aframomum corrorima *(Braun) Jansen are among the main cash crops. In some highland areas, the Bench cultivate *Hordeum vulgare *L., *Triticum *spp., beans, peas and *Eragrostis tef *(Zucc.) Trotter. They also raise cattle, sheep, goats, equine and poultry (unpublished data, Bench District Administration Office).

## Materials and methods

### Description of the study area

**Figure 1:**
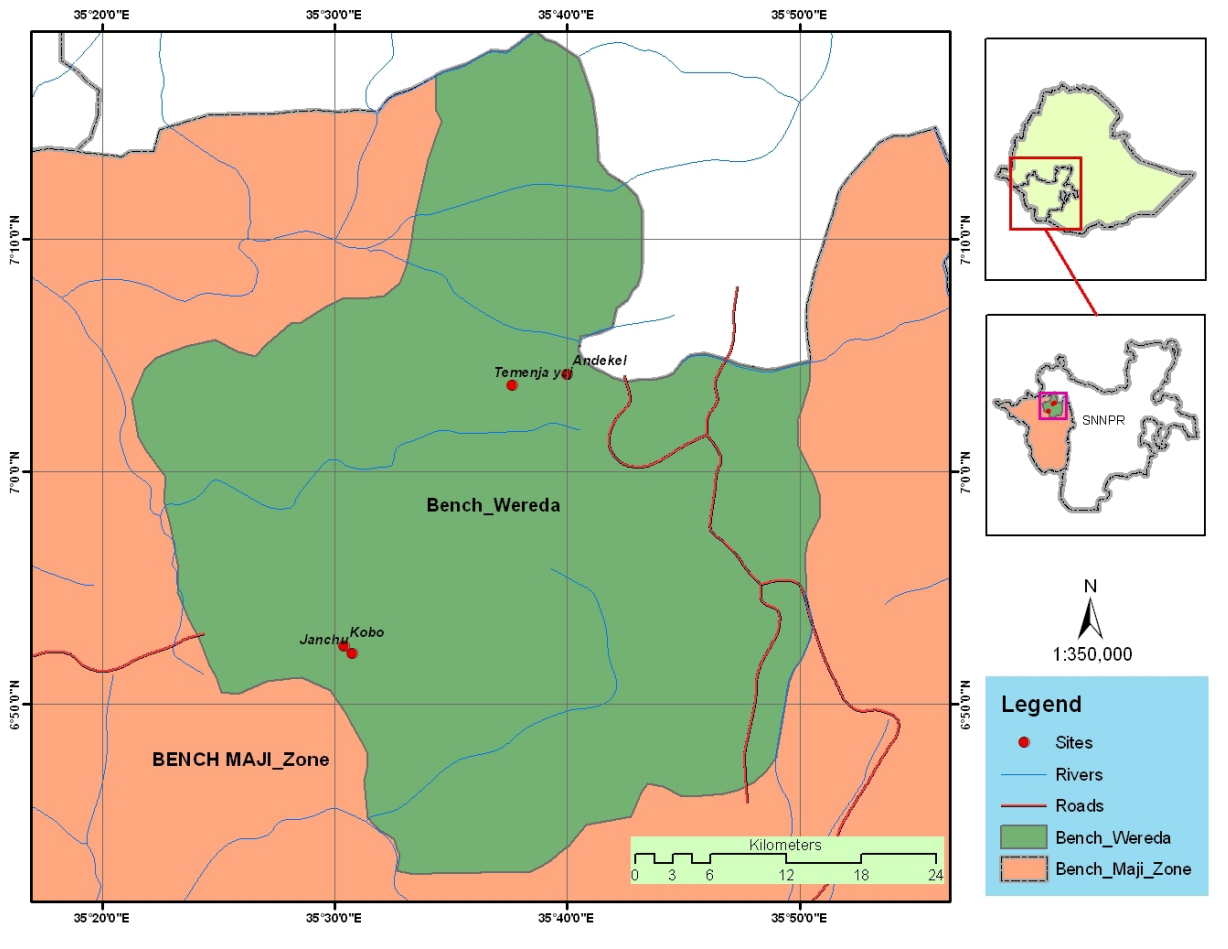
**Map of Bench District showing the study sites**.

The study was conducted in the Bench District where the Majority of the Bench people reside (Figure 1). According to the 1994 national census report [3], the human population of the District is 208, 339, the majority of which belonging to the Bench ethnic group. The Bench District has a total area of 212, 891 ha [4] and is divided into 55 rural and six town kebeles. Kebele is the lowest administrative unit in Ethiopia. The district receives an annual rainfall ranging between 2111 mm and 2563 mm [5]. The District covers both highland and semi-highland areas with altitudes ranging from 1500 to 2500 m a.s.l. There are spots of dense forests in the District that have been granted the status of high forests. Malaria, respiratory tract infections, intestinal parasites, skin-related diseases and typhoid fever are the major human health problems in the District (unpublished data, Bench-Maji Zone Health Office). One hospital, eight clinics and five health posts are available in the District. Blackleg, pasteurellosis, trypanasomiasis and intestinal parasitic infections are diseases of major veterinary importance in the District (unpublished data, Bench-Maji Zone Agriculture Office). Currently, there are two veterinary clinics and 12 animal health posts in the District.

### Selection of study sites and informants

For the ethnobotanical study, purposive sampling methods was employed to select a total of four kebeles from the Bench District located at varying distances from main road/modern healthcare centre. The kebeles were Temenja Yaj, Andekel, Janchu and Kob. Temenja Yaj and Andekel are located at less than 1 km from main road/modern health care centres, where as Janchu and Kob are found at a distance between 6 and 8 km from main road/modern healthcare facilities.

Informants, from the age of 18 and above and willing to sit for an interview, were sampled during transect walks made to houses in the selected kebeles. Quota sampling method was employed in the selection of informants in such a way that 50% are males and 50% are females. For comparative purpose, 10 informants that were identified as knowledgeable by local residents were also interviewed. Oral consent was sought from each informant before the start of the interviews.

### Methods of data collection

Ethnobotanical data were gathered between April 2004 and October 2006 mainly through individual interviews with selected informants using a semi-structured interview format [6-8]. Interviews were largely conducted in Bench language with the help of a local translator and responses were recorded in English.

During interview with each informant, information regarding the type of ailments (of human and livestock) managed, local names of the medicinal plants used by the Bench ethnic group against the reported ailments, the plant parts used, ways of remedy preparations, route of administration and dosage was gathered. Ethnobotanical data related to habitat and abundance, threat and local marketability of claimed medicinal plants as well cultivation practice of the Bench people were also collected. Specimens for most of the reported medicinal plants were collected, dried, properly identified and vouchers deposited at the National Herbarium of the Addis Ababa University.

### Data analysis

Data were organised and analysed with the help of Microsoft Excel spreadsheet software [9]. The presence or absence of significant differences between averages (at 95% confidence level) were checked using one-way analysis of variance (ANOVA) with the assistance of the software MINITAB Release 10.2 [10].

Fidelity level (FL) value was calculated for each medicinal plant reported to be used against human ailments to estimate its relative healing potential for specific major purposes by using the formula FL = I_p_/I_u _× 100, where I_p _is the number of informants who independently indicated the use of a species for the same major ailment and I_u _the total number of informants who mentioned the plant for any major ailment [11]. Prior to the calculation of FL, all human ailments mentioned during interviews were grouped into major disease categories by using a similar approach employed elsewhere [12]. It is assumed that plants which are used in some repetitive fashion for the same purpose are more likely to be biologically active [13].

Relative Importance (RI) value, a measure of diversity of medicinal application, was estimated for each claimed medicinal plant using the formula RI = NP +NCS [14]. NP is calculated by dividing the number of properties (in this case, specific ailments treated) attributed to a species divided by the total number of properties attributed to the most versatile species (species with the highest number of properties). NCS is computed by dividing the number of body systems (ailment categories) treated by a given species by the total number of body systems treated by the most versatile species. The highest possible RI value for a given species is 2.

## Results and discussion

### Medicinal plants reported

The study revealed 35 Bench medicinal plant species that belonged to 25 families and 34 genera (Table 1). Of the medicinal plants, 27 (77%) were herbs, five (14%) were trees, two (6%) were shrubs and one (3%) was a climber. The high usage of herbs among the Bench people could be an indication of their abundance as it was witnessed during visits to the study sites that areas very close to houses were well covered with herbs. The study area remains humid for most months of the year creating a favorable condition for the growth of herbs. The common use of herbaceous medicinal plants was also reported in studies carried out elsewhere in Ethiopia [15-21] and other parts of the world [22-24]. Asteraceae and Lamiaceae were represented by four species each, Apiaceae by three species, and Fabaceae and Polygonaceae by two species each. The rest of the families were represented by one species each. Asteraceae and Lamiaceae are among the most represented dicotyledonous families in the Flora of Ethiopia and Eritrea containing 440 [25] and 170 [26] species, respectively.

**Table 1:** Medicinal Plants of the Bench people

Scientific name	**Voucher no**.	Local plant name	Habit	Ailment treated^1^, local name given in bracket	Part used	Application route
*Aframomum corrorima *(Braun) Jansen [Zingiberaceae]		orsha	herb	stomachache (gobkin koshki)	seed	oral
*Ajuga integrifolia *Buch.-Ham. Ex D.Don [Lamiaceae]	MG-B32-2005	-	herb	retained placenta (kochiwutergu shida)	leaf	oral
*Anarrhinum forskaohlii *(Gmelin)	MG-B13-2005	turtsay	herb	boil (kursi)	Leaf	Topical
Cufodontis [Scrophulariaceae]				arthritis (pits)	leaf	topical
*Asparagus setaceous *(Kunth) Jessop [Asparagaceae]	MG-B3-2005	kingkang	herb	herpes (meshmesh)	cladode	topical
*Bidens pilosa *L. [Asteraceae]	MG-B14-2005	gurday	herb	retained placenta (kochiwutergu shida) ear infection (ay fug)	leaf/seed leaf	oral local (ear)
*Carica papaya *L. [Caricaceae]		papaya	tree	malaria (weba)	leaf	oral
*Centella asiatica *(L.) Urban [Apiaceae]	MG-B50-2005	tikus-asht	herb	Meningitis (tikus)	leaf	oral, topical
*Commelina benghalensis *L. [Commelinaceae]	MG-M88-2006	-	herb	skin infection (danch)	leaf	topical
*Cynoglossum amplifolium *Hochst. ex DC. [Boraginaceae]	MG-B4-2005	ziyad	herb	eye infection (tseskin)	Leaf	local (eye)
				cataract^2 ^(tsesin)	leaf	local (eye)
				wound (danch)	leaf	Topical
*Dicrocephala integrifolia *(L.f.) O.Ktze. [Asteraceae]	MG-B80-2005	-	herb	cataract (tsesin)	leaf	local (eye)
*Dyschoriste nagchana *(Nees) Bennet [Acanthaceae]	MG-B5-2005	kursi-asht	herb	boil (kursi)	leaf/whole/above ground	topical^3^
*Drymaria cordata *(L.) Schultes [Caryophyllaceae]	MG-B10-2005	pits-asht	herb	Arthritis (pits)	leaf	topical
*Hydrocotyle mannii *Kook.f. [Apiaceae]	MG-B29-2005	bezem-pesh	herb	cataract^4 ^(tsesin)	leaf	local (eye)
*Indigofera spicata *Forssk.	MG-B33-2005	kursi-asht	herb	boil (kursi)	above ground	oral
[Fabaceae]				tinea nigra (gayt)		topical
				meningitis^5 ^(tikus)	leaf	oral, topical
				meningitis (tikus)	above ground whole part	topical
*Ipomoea batatas *(L.) Lam. [Convolvulaceae]	MG-B7-2005	siquar-dinich	herb	boil^6 ^(kursi)	leaf	topical
*Leucas deflexa *Hook.f. [Lamiaceae]	MG-B8-2005	qechemen	herb	diarrhoea (shote) child diarrhoea (goken kosh) ascariasis (wesfat)	leaf above ground leaf	oral local (nose) oral, nasal
*Microglossa pyrifolia *(Lam.) O.Ktze. [Asteraceae]	MG-B37-2005	tikus-asht	shrub	meningitis^7 ^(tikus) cow mastitis	root/leaf/above ground leaf	oral, topical oral
*Ocimum lamiifolium *Benth. [Lamiaceae]	MG-B81-2005	michi-asht	herb	MICHI	leaf	oral, topical, local (eye)
*Pentas lanceolata *(Forssk.) Deflers [Rubiaceae]	MG-B6-2005	tigoch	herb	lymphadenitis (charush)	root, leaf	topical, oral
				boil (kursi)	leaf/flower	topical
				meningitis (tikus)	leaf/root	oral, topical
				abdominal cramp	root	oral
				(sheskan gazke)		
				arthritis (pits)	root/leaf	oral, topical
				cow mastitis	root, leaf	oral, topical
				meningitis (tikus)	leaf	oral, topical
*Phyllanthus pseudoniruri *Muell.Arg. [Euphorbiaceae]	MG-B28-2005	saz-asht	herb	herpes (meshmesh)	leaf	topical
*Phytolacca dodecandra *L'Hérit. [Phytolaccaceae]	MG-B82-2005	irtsets	shrub	dog rabies rabies	root leaf	oral oral
*Pouzolzia parasitica *(Forssk.) Schweinf. [Urticaceae]	MG-B18-2005	charun-asht	herb	lymphadenitis (charush)	leaf	oral, topical
*Prunus africana *(Hook.f.) Kalkm. [Rosaceae]	MG-B17-2005	omo	tree	ear infection (ay fug) toothache (bakin pug)	stem bark stem bark	local (ear) local (tooth)
*Ranunculus multifidus *Forssk. [Ranunculaceae]	MG-B9-2005	chadera	herb	cataract (tsesin) eye infection (tsesin)	Leaf leaf	local (eye) local (eye)
*Ritchiea albersii *Gilg [Capparidaceae]	MG-B42-2005	tikus-asht	tree	meningitis (tikus)	leaf	topical
*Rumex abyssinicus *Jacq. [Polygonaceae]	MG-B40-2005	danch-asht	herb	itching skin (danch)	root	topical
*Rumex nepalensis *Spreng. [Polygonaceae]	MG-B19-2005	germach	herb	abdominal cramp	whole	oral
				(sheskan gazke)		
				child diarrhoea	root	oral
				(goken kosh)		
				toothache (bakin	root	local (tooth)
				pug)		
				liver disease	root	oral
				(shorkeiz fuga)	root	local (eye)
				eye infection (tsesin)		
*Salvia nilotica *Juss ex Jacq. [Lamiaceae]	MG-B2-2005	ziyad	herb	MICHI^8^	leaf	nasal, topical (face), local (nose)
*Saniculata elata *Buch.-Ham ex D. Don [Apiaceae]	MG-B27-2005	kursi-asht	herb	boil (kursi)	above ground	topical
*Smilax anceps *Willd. [Smilacaceae]	MG-B30-2005	yarep	climber	ear infection (ay fug)	root	local (ear)
*Solanum anguivai *Lam. [Solanaceae]	MG-B12-2005	ambu	herb	Lymphadenitis (charush)	leaf	topical
*Trichilia dregeana *Sond. [Meliaceae]	MG-B83-2005	desh	tree	tinea capitis (gayt)	leaf	topical
*Trifolium rueppellianum *Fresen. [Fabaceae]	MG-B36-2005	tikus-asht	herb	meningitis (tikus)	whole part	oral, topical
*Verbena officinalis *L. [Verbenaceae]	MG-B84-2005	-	herb	stomachache (gobkin koshki)	root	oral
*Vernonia amygdalina *Del. [Scrophulariaceae]	MG-B1-2005	jampu	tree	MICHI^9^	leaf	topical (face), local (nose)

^1 ^Unless specified, the given disease is that of humans

^2 ^Plant mixed with *Hydrocotyle mannii*

^3 ^Plant mixed with *Ipomoea batatas*

^4 ^Plant mixed with *Cynoglossum amplifolium*

^5 ^Plant mixed with *Microglossa pyrifolia*

^6 ^Plant mixed with *Dyschorste nagchana*

^7 ^Plant mixed with *Indigofera spicata*

^8 ^Plant mixed with *Vernonia amygdalina*

^9 ^Plant mixed with *Salvia nilotica*

### Ailments treated, plant parts used and modes of remedy preparations

Of the Bench medicinal plants, 32 (91%) were used against human ailments and three (9%) to treat both human and livestock ailments (Table 1). The Bench used the highest proportions of medicinal plants to treat three human ailments: skin-related problems (46%), eye problems (20%) and meningitis (20%). Some were used against the following eight human ailments: gastro-intestinal complaints (14%), ear diseases (11%), deformation of fingers (9%), MICHI (6%), toothache (6%) rheumatic pain (3%), rabies (3%) and jaundice (3%). MICHI is a local term characterized by fever, headache and sometimes lip sores. Of the three plants used for veterinary purposes, two were used to treat mastitis and one against rabies. The fact that higher proportions of medicinal plants are used by the Bench people to treat skin-related ailments could be attributed to the high prevalence of the disease in the area. Skin-related disorder is one of the major public health problems in Bench District (unpublished data, Bench-Maji Zone Health Office).

The majority (71%) of the Bench medicinal plants were sought for their leaf part. The common use of leaf in the preparation of remedies could partly be due to the relative ease of finding this plant part. Leaves remain green and plenty for most months of the year since the area in which the Bench people reside receives good rainfall for about eight months in the year. The use of leaves in the preparation of remedies is also common elsewhere [16,22,23,27-31]. Most remedies were prepared and used immediately after harvest: 86% were processed while fresh and 3% were prepared after a quick drying. Eleven percent of the Bench remedies were reported to be dried and stored for future use. The frequent use of freshly processed remedies could indicate the availability of copious plant materials in the vicinity to be picked any time. Other studies conducted elsewhere also indicated the wider use of fresh materials [20,21,27,31,32]. The frequent use of fresh materials might also be an attempt not to lose volatile oils, the concentration of which could deteriorate on drying.

Eighty one percent of the Bench remedies were prepared in juice or paste form. Remedies were seldom prepared as poultice (6%), decoction (4%), powder (4%), infusion (3%) or used unprocessed (1%). Fifty percent of the Bench remedies were prepared with out the use of diluents, while 40% were prepared with the addition of water. Other diluents used in the preparation of remedies included coffee (6%), milk (3%) and human saliva (1%). Water was the most frequently used diluent in the preparation of remedies. Availability could be one of the criteria used in the selection of diluents.

### Route of administration and dosage

Thirty nine percent of the Bench remedies were applied on the skin while 33% were taken orally and 16% administered through the eyes. Few remedy preparations were taken nasally (4%), auricularly (4%) or applied locally in the mouth (3%). The fact that most Bench remedies are applied topically on the skin could be attributed to the high prevalence of skin-related ailments in the study area. Most treatments were reported to be completed within one or two days; most of them taken once a day. Liquid remedies administered to humans were usually measured by tea or coffee glasses or plastic cups, or number of drops. When patients did not show any sign of recovery from their illnesses after treatment completion, they turned to a nearby modern health centres.

### Marketability, habitat and abundance of medicinal plants

Most Bench medicinal plants were not available for sale at local markets; only three (*Aframomum corrorima*, *Carica papaya *and *Ipomoea batatas*) were reported to be sold at local markets but mainly for their uses as spice or food. Medicinal plants were freely harvested by users from the immediate environment in which they were abundantly found. Other studies conducted elsewhere in Ethiopia, however, indicated a wide domestic trade of medicinal plants [33-36]. In contrast to some developing countries, there is no official report, so far, of any medicinal plant export from the country.

The majority (86%) of Bench medicinal plants were uncultivated species, most of them weeds abundantly growing in disturbed habitats, mainly in crop fields, fallowlands and along hedgerows. Their abundance might even increase in future due to agricultural expansion and rising private investment in the area. Five were reported to be planted by users in homegardens and cultivation fields, of which only two (*Ranunculus multifidus *and *Pouzolzia parasitica*) were cultivated for their sole medicinal values. The other three (*Aframomum corrorima*, *Carica papaya *and *Ipomoea batatas*) were grown primarily for other purposes (spice and food). A study conducted in the rural Bahir Dar Zuria district, Northwestern Ethiopia [37] also showed the common use of weeds for medicinal purposes. Similarly, a study carried out in Pernambuco State, Brazil [38], indicated the frequent use of weeds by herbalists, harvested from back yards and small farms. It was stated that weeds are often abundant near at hand, easy to harvest and are frequently rich in bioactive compounds and as a result they are amply represented in contemporary tropical healing floras [39]. The abundance of one Bench medicinal plant (*Prunus africana*) was, however, reported to be rapidly declining from time to time due to selective cutting and/or deforestation. In the study area, *Prunus africana *is frequently felled to be used for house construction and as firewood. Its wood is also frequently used to make pestle. In Cameroon, *Prunus africana *has been made scarce because of excessive harvest of its bark for international market. Annually, more than 3,000 tones of the bark of *Prunus Africana *is exported from Africa to Europe for its use to treat benign prostatic hypertrophy, of which nearly two-third is harvested in Cameroon [40].

### Informant consensus

Of the Bench medicinal plants reported against human ailments, *Dyschoriste nagchana *(against cutaneous/subcutaneous disease), *Microglossa pyrifolia *(against meningitis) and *Salvia nilotica *(against MICHI) were the ones with highest fidelity level (FL) values, each scoring 100% (Table 2), which could be taken as indication of the good healing potential of the plants. It is assumed that plants that are used in a repetitive fashion are more likely to be biologically active [13].

**Table 2:** FL values for Bench medicinal plants cited by three or more informants for being used against same ailment category

*Medicinal plant*	*Ailment category*	*I*_*p*_	*I*_*u*_	*FL value (%)*
*Dyschoriste nagchana*	cutaneous/subcutaneous diseases	5	5	100
*Microglossa pyrifolia*	meningitis	3	3	100
*Salvia nilotica*	MICHI	3	3	100
*Ranunculus multifidus*	eye disease	5	6	83
*Leucas deflexa*	gastro-intestinal complaints	3	4	75
*Ocimum lamiifolium*	MICHI	3	4	75
*Cynoglossum amplifolium*	eye disease	3	4	75

### Medicinal plants and their diversity of use

*Pentas lanceolata *was the Bench medicinal plant with the highest relative importance (RI) value (2.0), followed by *Rumex nepalensis *(1.8) (Table 3). *Pentas lanceolata *was recommended against abdominal cramp, meningitis, boil, lymphadenitis and cattle mastitis. *Rumex nepalensis *was used to treat eye infection, abdominal cramp, childhood diarrhoea, liver disease and toothache. High RI value could partly be a reflection of abundance as medicinal plants that are found in plenty in a given area are more likely to be favoured for remedy preparation than those that are encountered rarely. High versatility of medicinal plants could also indicate higher diversity of active compounds contained by the species.

**Table 3:** RI values for Bench medicinal plants used against three or more specific ailments

*Scientific name*	*NP*	*NCS*	*Relative importance (RI) values*
*Pentas lanceolata*	1	1	2.0
*Rumex nepalensis*	1	0.8	1.8
*Indigofera spicata*	0.6	0.6	1.2
*Cynoglossum amplifolium*	0.6	0.4	1.0
*Ranunculus multifidus*	0.6	0.4	1.0

### Medicinal plant knowledge between different social groups within the Bench community

The study indicated that significantly higher average numbers of medicinal plants (p < 0.05) were reported by men (mean = 1.0 ± 0.002) than women (mean = 0.3 ± 0.0001), by informants above 40 years of age (mean = 1.5 ± 0.002) than those belonging to an age group of 18 to 40 years (mean = 0.5 ± 0.0001) and by illiterate (mean = 1.3 ± 0.001) than literate ones (mean = 0.5 ± 0.0001). Significantly higher average number of plants (p < 0.05) was also reported by informants identified as knowledgeable by locals (mean = 2.1 ± 0.001) than by people who were randomly picked during visits made to houses in the selected kebeles (mean = 0.5 ± 0.0001).

The fact that men have better medicinal plant knowledge than women could be due to the reason that boys are usually favoured in the transfer of the knowledge. Other studies conducted elsewhere demonstrated similar results [16,41,42]. However, a study conducted in the rural Bahir Dar Zuria district, Northwestern Ethiopia [37], demonstrated that there is no significant difference in medicinal plant knowledge between men and women.

The vertical transfer of medicinal plant knowledge among the Bench community is not taking place in a proper manner due to the lack of interest among the younger generation to learn and practice it, which might be attributed to the ever increasing influence of modernization. Results of studies conducted elsewhere also revealed similar findings [24,37,43,44]. Literate people in the study area were found to have less knowledge of medicinal plants as compared to illiterate ones as the former are more likely to be exposed to modernization as also revealed by studies conducted elsewhere [43,45].

Significant difference was not observed (p > 0.05) in medicinal plant knowledge between Bench informants residing at kebeles located at less than 1 km distance from nearby road/modern healthcare centres (mean = 0.6 ± 0.0002) and those inhabiting kebeles located at distances between 6 and 8 km from nearby road/modern healthcare centres (mean = 0.7 ± 0.0003). This might indicate that the 6-8 km distance of residential areas from roads/healthcare centres is not far enough to make residents dwell more on their traditional medical practice and as a result have better knowledge of the same as compared to those residing at a 1 km distance. However, results of other studies conducted elsewhere reported a positive relationship between distance from modern healthcare facilities and local knowledge [46-50].

### Medicinal plant Knowledge secrecy, mode of transfer and threats

The study revealed that most Bench remedies were prepared and administrated at a household level, which is in agreement to findings of other works [37,51]. When deemed necessary, people could seek the help of knowledgeable people in their respective locality with no or nominal charge. The majority (75%) of Bench informants reported that they kept their medicinal plant knowledge secret. They further revealed that free transfer of knowledge could only take place along the family line, usually from parents to sons. It was found out that transfer of knowledge to people outside the family circle could only take place on substantial payment. The secrecy of traditional medical practice is also a common phenomenon elsewhere in the country [20,52,53].

## Conclusion

This study showed that traditional medicine, mainly involving the use of medicinal plants, is playing a significant role in meeting the primary healthcare needs of the Bench ethnic group. Acceptance of traditional medicine and limited access to modern healthcare facilities could be considered as the main factors for the continuation of the practice.

The majority of Bench medicinal plants were herbs which could be attributed to their abundance in areas very close to houses as compared to trees or shrubs. Newly harvested plant materials are mostly used in the preparation of remedies indicating little practice by people to dry and store medicines for future uses, which is an indication of the availability of copious plant materials in the vicinity to be picked any time.

The Bench plants *Dyschoriste nagchana *(against skin diseases), *Microglossa pyrifolia *(against meningitis) and *Salvia nilotica *(against MICHI) were the ones with the highest fidelity level (FL) values, an indication of their high healing potential. Priority should, therefore, be given to these plants to test their efficacy and their toxicity.

The immediate and serious threat to the local medical practice in the study area seems to have come from the increasing influence of modernization. As there is no adequate modern healthcare service provision in the study area, loss of local medical knowledge and practice could negatively affect the healthcare system of the people. To arrest or slow down the trend, awareness on the contribution of traditional medical practice towards fulfilling the primary healthcare needs of the local people should be created among the youth.

## Competing interests

The authors declare that they have no competing interests.

## Authors' contributions

All authors had significant intellectual contribution towards the design of the study, data collection and analysis and write-up of the manuscript. All authors read and approved the final manuscript.
